# A conductive self healing polymeric binder using hydrogen bonding for Si anodes in lithium ion batteries

**DOI:** 10.1038/s41598-020-71625-3

**Published:** 2020-09-11

**Authors:** Jaebin Nam, Eunsoo Kim, Rajeev K.K., Yeonho Kim, Tae-Hyun Kim

**Affiliations:** 1 Organic Material Synthesis Laboratory, Department of Chemistry, Incheon National University, Incheon, South Korea; 2 Research Institute of Basic Sciences, Incheon National University, 119 Academy-ro, Songdo-dong, Yeonsu-gu, Incheon, 22012 South Korea

**Keywords:** Polymer characterization, Energy, Batteries

## Abstract

A ureido-pyrimidinone (UPy)-functionalized poly(acrylic acid) grafted with poly(ethylene glycol)(PEG), designated PAU-g-PEG, was developed as a high performance polymer binder for Si anodes in lithium-ion batteries. By introducing both a ureido-pyrimidinone (UPy) unit, which is capable of self-healing through dynamic hydrogen bonding within molecules as well as with Si, and an ion-conducting PEG onto the side chain of the poly(acrylic acid), this water-based self-healable and conductive polymer binder can effectively accommodate the volume changes of Si, while maintaining electronic integrity, in an electrode during repeated charge/discharge cycles. The Si@PAU-*g*-PEG electrode retained a high capacity of 1,450.2 mAh g^−1^ and a Coulombic efficiency of 99.4% even after 350 cycles under a C-rate of 0.5 C. Under a high C-rate of 3 C, an outstanding capacity of 2,500 mAh g^−1^ was also achieved, thus demonstrating its potential for improving the electrochemical performance of Si anodes.

## Introduction

High-capacity battery materials are in high demand for use in lithium-ion batteries (LIBs) in Electric Vehicles (EVs) and Energy Storage Systems (ESS), which have high energy density requirements. For such applications, silicon (4,200 mAh g^−1^, Li_4.4_Si), which has a significantly higher theoretical capacity than commercialized graphite (372 mAh g^−1^, LiC_6_), is recognized to be a promising anode material. In addition to its high capacity, silicon is an effective material for anodes because of its low discharge voltage, and is suited for batteries with high energy density. Silicon is also highly cost-effective being one of the most abundant elements on earth^1–6^.

Despite the aforementioned advantages, however, silicon exhibits huge (ca. 400%) volumetric expansion/shrinking during repeated charge/discharge processes, which causes Si-based LIBs (cells) to have generally poor cycle lives. These changes in volume not only tend to pulverize silicon particles, and lead to the repeated loss and formation of the solid electrolyte interphase (SEI) layer, but also cause detachment from the current collector and structural collapse between electrodes. Since these issues are associated with the loss of active material (Si), low Coulombic efficiency, and loss of contact for ionic and electrical conduction, they ultimately result in the rapid decay of cell capacity. As such, few Si-based electrodes are currently comprised entirely of Si, but are usually combinations of graphite and small amounts of Si^7–11^.

To address problems arising from the large volumetric expansion of Si, various approaches to develop nano-structured Si have been proposed. However, in practice, they have proven expensive to implement. More importantly, the accumulation of irreversible damage to the Si electrodes during repeated charge/discharge cycles has made it particularly difficult to improve Si-based cell performance^12–21^.

An alternative approach to mitigate the problems of Si volumetric expansion is to use polymeric binders as an essential component in cell manufacturing. For example, carboxymethyl cellulose (CMC), alginate, poly(acrylic acid) (PAA) and poly(vinyl alcohol) (PVA) which all interact strongly with Si particles, have been used as polymeric binders to enhance cycle life by relieving the physical stress caused by the volumetric expansion of silicon electrodes. The development of water-soluble polymers is also advantageous because it means environmentally-friendly water can be used in the electrode manufacturing process. In contrast, PVdF and other conventional polymeric binders require toxic and volatile organic solvents for electrode manufacturing^22–27^.

Other attempts have been made to form silicon electrodes with 3D networks using functional polymers such as grafted polymers and crosslinked polymers, to enhance their physical properties. Electrodes fabricated from these polymer binders are more effective than the above-mentioned linear type polymer binders. Another approach involves the use of either ionically or electrically conducting polymers to provide ion/electron pathways in the electrodes, respectively^28–37^. Although some progress has been made as a result of these efforts, none as yet have been able to repair the structural damage in the Si electrode caused by the large volume change of Si. This continues to be an obstacle to improving cell performance.

Against this backdrop, studies have recently been reported which use self-healable supramolecular polymers as binders for silicon anodes. Since these binders engage in reversible intermolecular interactions that enable self-healing, they can effectively accommodate the volumetric expansion of Si, maintaining an electrical contact with the active material, conductive agent and current collector, and thus contribute to capacity retention and cycling stability^38–43^. In other words, noncovalent polymer networks, which are reversible, are expected to be better at accommodating volumetric expansion, compared to covalent polymer networks, which are irreversible^44–46^.

The ureido-pyrimidone (UPy) molecule is well-known to exhibit self-healing properties as a result of strong hydrogen bonding^47–50^. Recently, a few polymers containing the ureido-pyrimidinone (UPy) molecule have been reported as binders for silicon anodes^41,43^. These studies have demonstrated the ability of self-healing polymers to form reversible noncovalent supramolecular polymer networks in Si electrodes, effectively mitigating the physical stresses caused by Si volume changes and hence improving the electrochemical performance of the corresponding electrodes. However, the polymer binders used in these reports have factors that limit their practical application, either because a toxic organic solvent is required to fabricate the electrodes due to the poor solubility of the binder^41^ or the electrode cycle life was short^43^.

**Figure 1:**
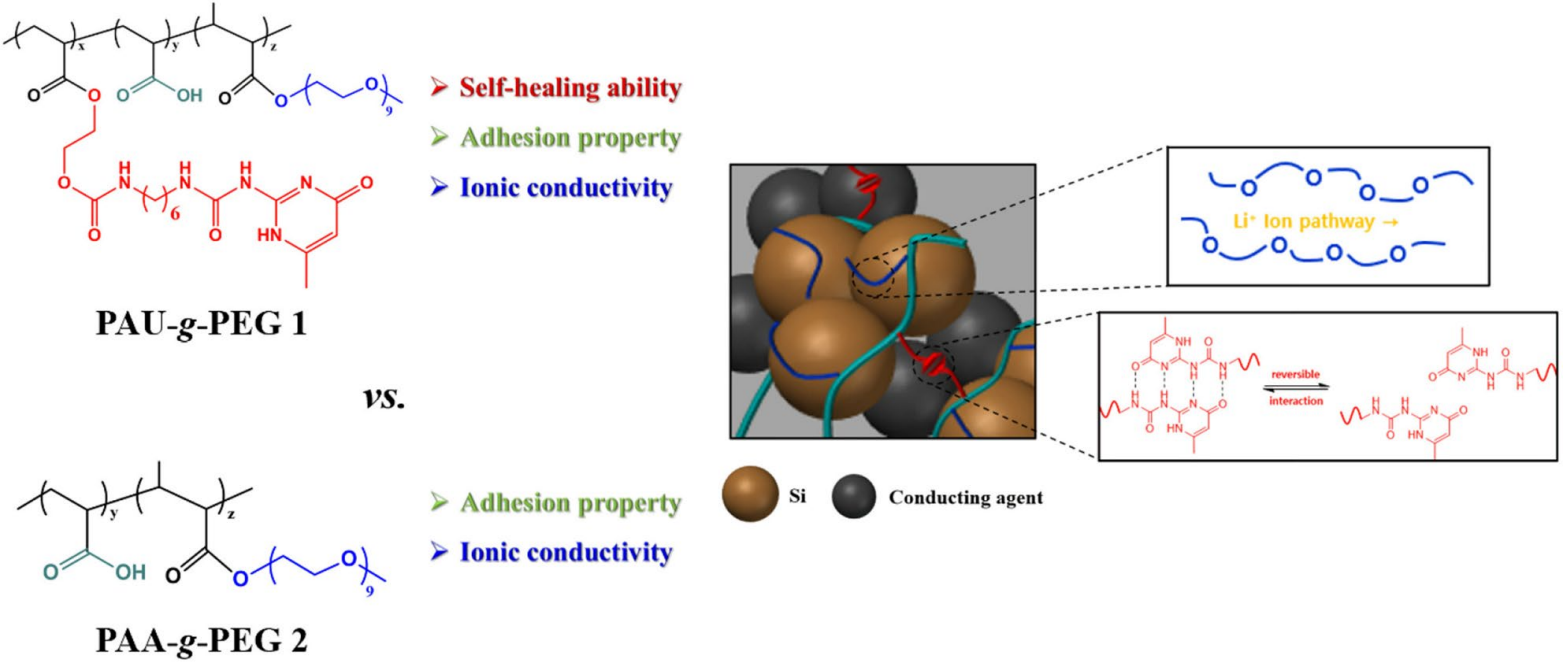
Schematic illustrations of the polymeric binder, PAU-*g*-PEG **1**, with both reversible self-healing and Li^+^-conductive properties, compared with PAA-g-PEG **2** with only Li^+^-conductive sites, for silicon electrodes.

In this study, we developed a new multi-functional binder based on a self-healable polymer where ionic conductivity is further assigned, to be used for high-performance silicon electrodes with stable capacity retention even under high current density. To accomplish this, a Ureido-pyrimidinone (UPy) was partially introduced on poly(acrylic acid), PAA. UPy has fast self-healing through dynamic H-bonding within molecules, and interacts strongly with silicon particles and the oxidized surface (copper oxide) of the current collector. Poly(ethylene glycol) (PEG), which can effectively transfer lithium ions by coordinating with lithium ions using their lone-pair electrons on the oxygen of the ether groups^51–53^, was further grafted onto this UPy-functionalized PAA as an ion-conducting group, thereby forming poly(acrylic acid-*co*-UPy-acrylate)-*grafted*-PEG, PAU-*g*-PEG **1** (Fig. 1). PAA was selected as the main polymer because it is a water-soluble polymer capable of interacting with silicon particles.

Furthermore, the self-healable UPy molecules are expected to crosslink between the polymer chains as well as with Si particles to form a more stable 3D network, which will allow it to accommodate the volume expansion of Si more effectively. The self-healable property of the PAU-*g*-PEG polymer binder can also efficiently restore structural deterioration which has occurred during the charge/discharge process, while maintaining the physical stability of the electrodes.

By sequentially introducing functional groups with self-healing ability and ionic conductivity to the PAA main chain, the compositions of the functional groups were controlled, achieving optimal properties, while the water-soluble nature of the polymeric binders was maintained. We also investigated how such functional groups affected the physical properties of the binders, and the electrochemical properties of the corresponding Si-based cells.

## Results

### Characterization of poly(acrylic acid-*co*-UPy acrylate)-*grafted*-PEG, PAU-*g*-PEG 1 and poly(acrylic acid)-*grafted*-PEG (PAA-*g*-PEG) 2

^1^H-NMR and FT-IR spectroscopic analysis was employed for the structural analysis of PAU-*g*-PEG **1** and the control polymer PAA-*g*-PEG **2** (Figure S2 and S3).

The peak observed for copolymer **1** near 12.2 ppm was for carboxylic acid (–COOH^1^), which indicated that the tBA groups had undergone hydrolysis successfully. The three peaks at 7.45, 7.04, and 5.72 ppm were due to the protons of the UPy molecules (namely, H^2^, H^3^, H^4^), and this showed that the monomer including UPy was successfully copolymerized for copolymer **1**^47^. The peaks near 3.48 ppm were for the protons in the PEG copolymers (H^5^), demonstrating that PEG had been grafted onto the polymers. Lastly, the peaks around 1–3 ppm were for the protons of the alkyl chains (H^6^, H^7^, H^8^) in the copolymer backbone. The ratio of UPyA:acrylic acid:PEG-MEMA in the PAU-*g*-PEG copolymer **1**, that is x:y:z, was calculated to be 1:70:1.6 based on the comparative integral ratio of the proton peaks (H^1^:H^2,3,4^:H^5^). This ratio was slightly different from the feed ratio used in the reactions, suggesting different reactivity between the comonomers (Table S1).

The PAA-*g*-PEG copolymer **2**, prepared as a control, was also analyzed by ^1^H-NMR spectroscopic analysis, and the ratio of acrylic acid to PEG-MEMA was calculated from the integral ratio of proton peaks of carboxylic acid (H^1^) and PEG (H^1^) to be 70:1.6 (that is, y:z), the same as the ratio of acrylic acid to PEG-MEMA in PAU-*g*-PEG **1** (Figure S2 and Table S1).

The FT-IR spectra of PAU-g-PEG **1** and PAA-g-PEG **2** were further analyzed. The peak at 1758 cm^−1^ (C=O stretching) in the ester group was observed, together with the broad peak at 2,500–3,500 cm^−1^ (O–H stretching) and peak at 1705 cm^−1^ (C=O stretching) originated from the carboxylic acid group of PAA, confirming the successful grafting of both UPy and PEG unit onto PAA (Figure S3).

### Thermal stability of the polymeric binder

The thermal decomposition behaviors of the synthesized copolymers **1**, **2** and PAA were confirmed using the TGA analysis (Figure S4). All three exhibited similar decomposition behavior because they had the same PAA unit as the main chain. The first degradation of PAU-*g*-PEG, PAA-*g*-PEG and PAA was at 180–322 °C (weight loss of 22 wt%), 179–322 °C (weight loss of 21 wt%) and 184–280 °C (weight loss of 9 wt%). This weight loss corresponded to dehydration, forming PAA anhydrides. In case of PAU-*g*-PEG and PAA-*g*-PEG, decomposition of both UPy and PEG units grafted onto the polymer side chain additionally occurred, causing a higher weight loss^54^. All three binders (PAU-g-PEG, PAA-g-PEG and PAA) showed high thermal stability up to about 180 °C.

### Mechanical properties of the polymeric binders used for silicon electrodes

**Figure 2:**
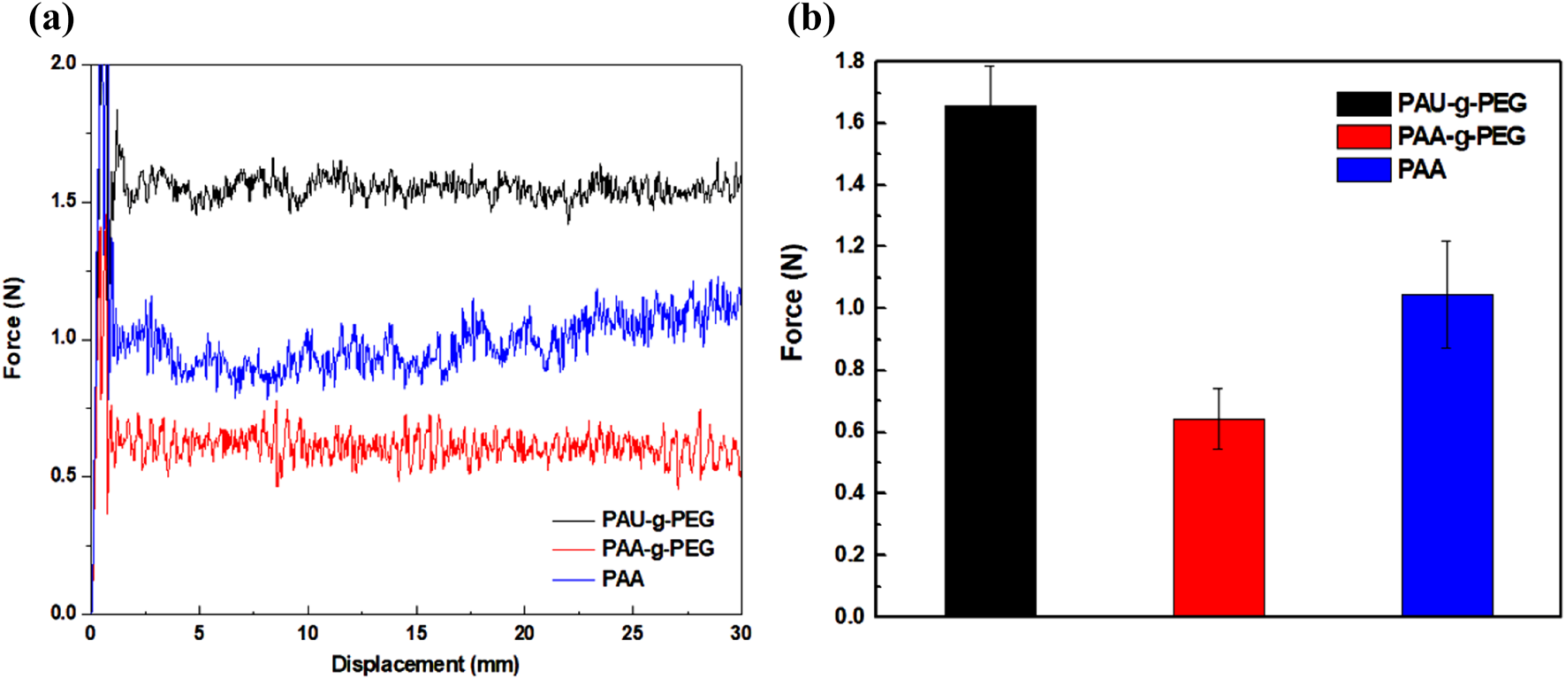
Adhesive properties of the silicon electrodes using PAU-*g*-PEG, PAA-*g*-PEG and PAA as polymeric binders: (**a**) force–displacement curves and (**b**) peel strength graphs.

An 180° peel-off test of the Si electrode made with the newly developed polymer binders was conducted to determine the physical stability, that is the adhesive force, of PAU-*g*-PEG **1**, which was designed to have both self-healing ability and ionic conductivity. The results were compared to that of pristine poly(acrylic acid) (PAA) with a molecular weight (*M*_*w*_ = 100,000 g mol^−1^) similar to that of copolymers **1**, and also with PAA-*g*-PEG **2**, which has Li^+^-conductivity but no self-healing sites (Fig. 2a). All three electrodes had the same weight ratio (SiNP:Super P:binder = 6:2:2) and controlled thickness.

The results showed that the adhesive force of the polymers was in the order of PAU-*g*-PEG > PAA > PAA-*g*-PEG. The PAA itself had relatively high adhesion due to its interactions with the hydroxyl (–OH) functional groups on the silicon surface and the oxidized surface of the current collector. The adhesive force of copolymer PAA-*g*-PEG **2**, prepared by grafting just PEG onto the PPA main chain, was slightly lower than PAA. This can be attributed to the weakened interactions among electrode components in the presence of PEG, which engages fewer hydrogen bonds compared to the PAA copolymer.

The PAU-*g*-PEG copolymer which was comprised of both PEG grafted onto the PAA main chain and the self-healing UPy molecules showed a high adhesive force of 1.6 N, significantly higher than that of PAA polymers, even with the grafting of PEG (Fig. 2b). The interactions between the electrode components were reinforced by the introduction of UPy, through strong H-bonding, and the UPy molecules further contributed to the enhanced physical properties of the polymers by serving as physical crosslinking sites between the polymer chains and also with Si particles, to form a more stable 3D network, as reported before^55^.

Ultimately, the PAU-*g*-PEG copolymer resolved the problem of weakened adhesive force caused by PEG grafting, by introducing UPy molecules, and it achieved higher physical stability than PAA. This enhanced the physical properties of the PAU-*g*-PEG copolymer binder, and was expected to accommodate the volume expansion of Si more effectively (as will be discussed later).

### Self-healing ability of the polymeric binder

**Figure 3:**
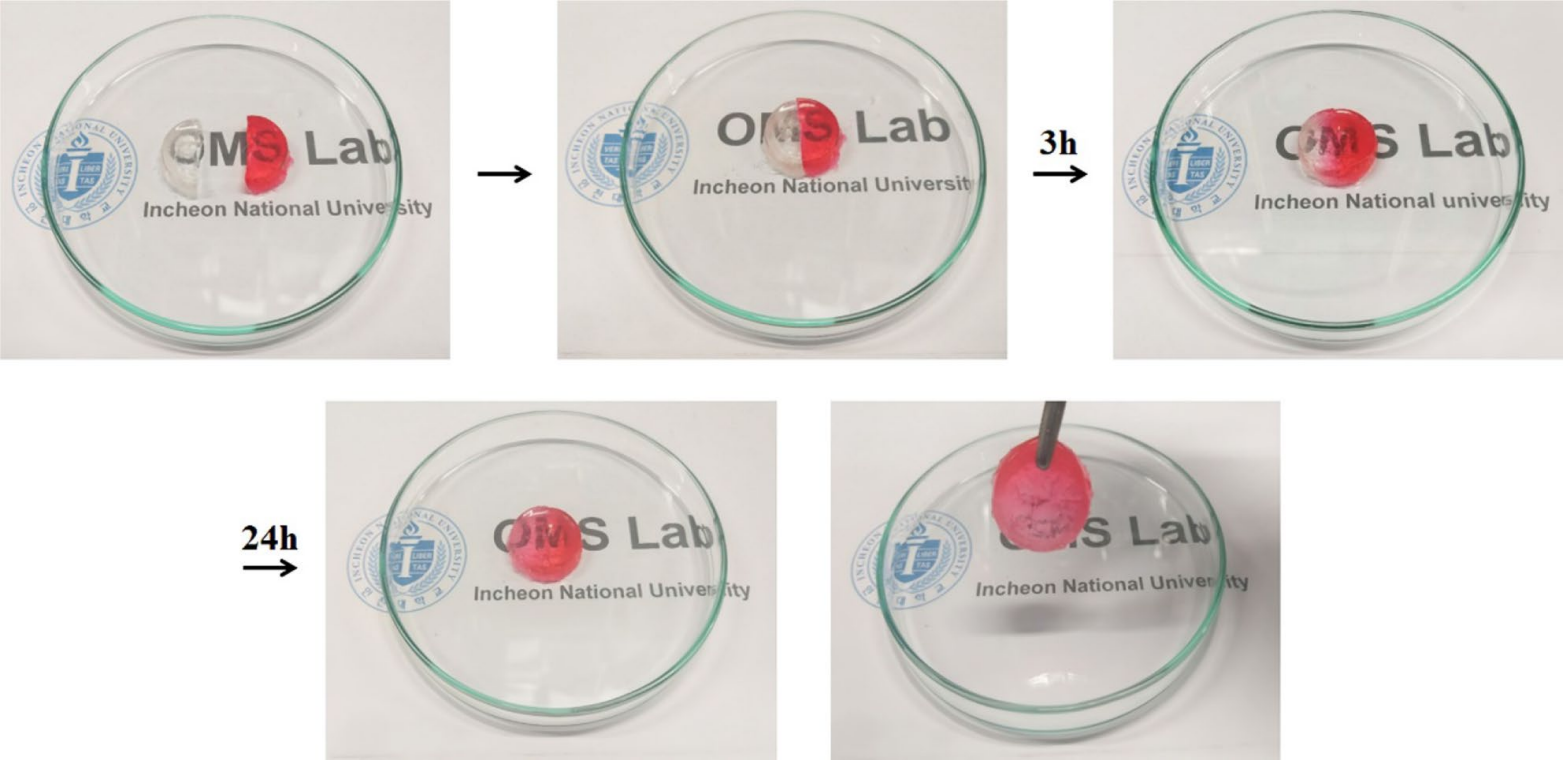
Optical images of the self-healing ability of the PAU-*g*-PEG-based hydrogel.

Next, we assessed the self-healing ability of copolymer PAU-*g*-PEG **1**, which had been found to significantly enhance the adhesive force of the electrodes through strong hydrogen bonds (Fig. 3). To this end, the synthesized copolymer **1** was firstly hydrogelated using the freeze/thaw method as follows. A 35 wt% PAU-*g*-PEG solution was prepared and hydrogelated by freezing for 1 h at − 20 °C and thawing for 24 h at room temperature (Figure S5a)^56^.

In contrast, the PAA 35 wt% solution formed by the same process did not retain its hydrogelated form after the freezing/thawing process, and returned to a liquid state (Figure S5b), suggesting that the UPy molecules of PAU-*g*-PEG contributed to its ability to remain in gel form.

Next, the self-healing ability of the copolymer PAU-*g*-PEG **1** was examined as follows.

The prepared PAU-*g*-PEG hydrogel was first cut into two pieces, one of which was dyed red to help illustrate the self-healing process. The pieces were stored at room temperature in contact with each other and without any special treatment. About 3 h later, it can be seen that the red dye gradually diffused from the surface of the cut hydrogel, and after about 24 h, the dye was completely spread and there was almost no trace of slicing activity. Overall, the self-healed hydrogel maintained the surface contact in a stable manner (Fig. 3).

Despite its low content of UPy molecules at 1.4 mol%, PAU-*g*-PEG **1** exhibited the characteristics of a self-healable supramolecular polymer, based on strong hydrogen bonds.

### Electrochemical cell performance of the silicon electrodes

**Figure 4:**
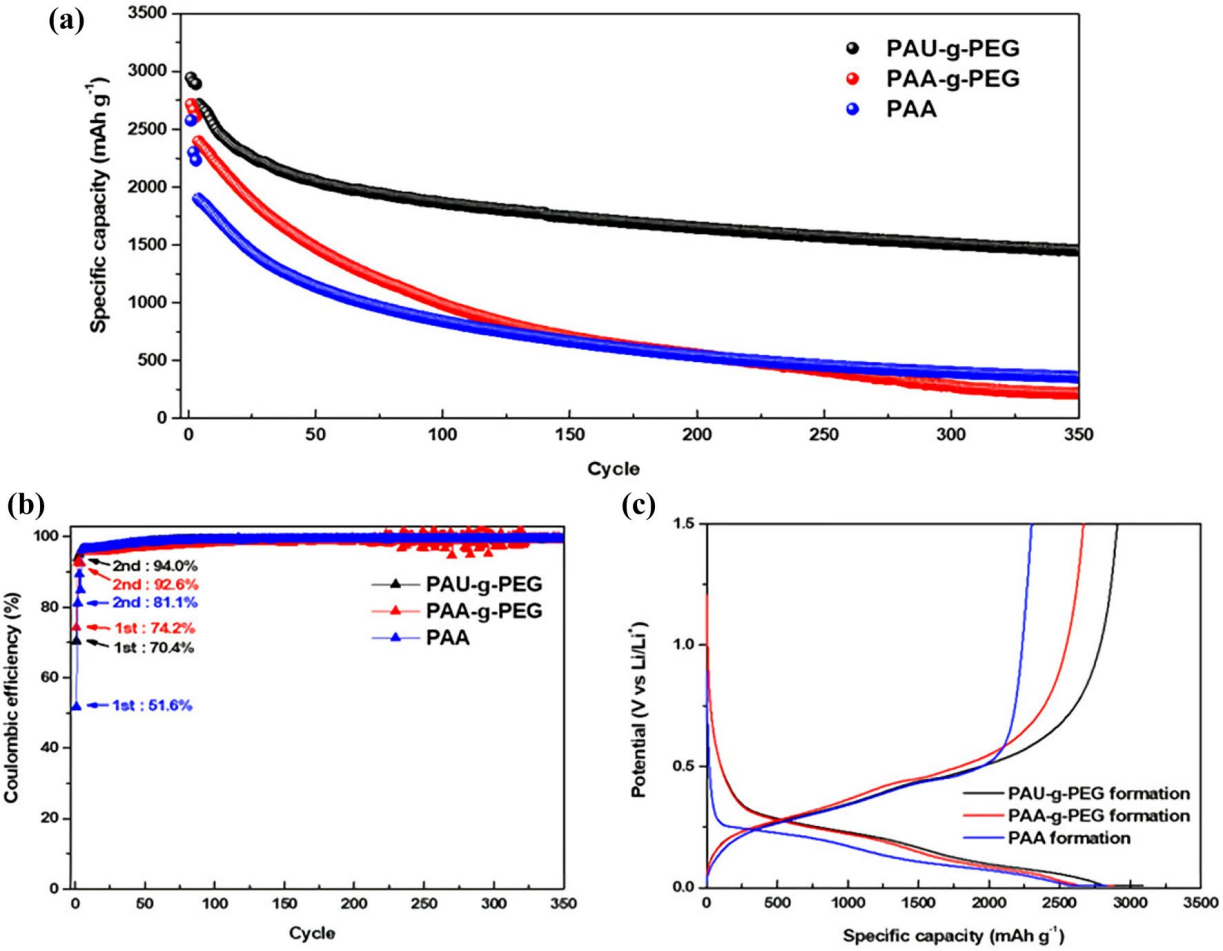
Electrochemical performance of the silicon electrodes using PAU-*g*-PEG, PAA-*g*-PEG and PAA as the polymeric binder: (**a**) the long-term cycling performance (@ 0.5 C), (**b**) the Coulombic efficiency, (**c**) the charge–discharge profiles at formation cycle (@ 0.1 C).

Silicon electrodes were fabricated using PAU-*g*-PEG and PAA-*g*-PEG copolymers as well as PAA as binders, and their electrochemical performance was assessed using both a coin cell and 3-electrode cell (Fig. 4). The coin cell, in the form of a half cell, was comprised of a silicon electrode as the working electrode, and a lithium metal disk as the counter and reference electrode. For the 3-electrode cell, a piece of lithium metal was used as the reference electrode.

To assess their long-term cycling performance during the charge/discharge process, three electrodes with a mass loading level in the range of 0.5–0.6 mg cm^−2^ were charged/discharged at a low current density of 0.1 C and a voltage range of 0.01–1.5 V for the initial formation cycles (3 cycles), and at 0.5 C in the following cycles (Fig. 4a).

The PAA-based electrode (Si@PAA) showed the lowest initial capacity (2,575.7 mAh g^−1^) and fast capacity fading in the early cycles, but the capacity was stabilized in later cycles.

The PAA-*g*-PEG-based silicon electrode (Si@PAA-*g*-PEG) showed a higher initial capacity (2,716.9 mAh g^−1^) than that of PAA, but also experienced severe initial capacity decay. The poorest capacity retention, with capacity dropping below that of even the PAA electrode after 200 cycles, was obtained for the Si@PAA-*g*-PEG electrode.

Meanwhile, the silicon electrode with the PAU-*g*-PEG binder (Si@PAU-*g*-PEG) displayed the highest initial capacity of 2,946.7 mAh g^−1^, and also showed the highest capacity retention among the three electrodes.

The capacity and capacity retention of the three silicon electrodes at 350 cycles were 1,450.2 mAh g^−1^, 53.3%; 356.6 mAh g^−1^, 18.8%; and 212.8 mAh g^−1^, 8.8% respectively, or PAU-*g*-PEG > PAA > PAA-g-PEG when arranged in increasing order. These results are consistent with the physical stability of the electrodes. The hydrogen bonding ability of PAA, together with the self-healing property of UPy, in the PAU-*g*-PEG electrode is believed to have effectively alleviated the structural deterioration caused by volumetric changes in the silicon during the charge/discharge process.

Moreover, both the PAU-*g*-PEG and PAA-*g*-PEG electrodes showed a higher initial capacity than the PAA electrode. This result is ascribed to the increase in ionic conductivity via the Li^+^ conductive pathway formed by grafting PEG in these two binders (to be discussed later). The PAU-*g*-PEG binder, in particular, was more physically stable than the other two binders, which was advantageous to the formation of the lithium ion pathway, and helped to achieve the highest initial capacity.

The initial Coulombic efficiency of the three binders was in the order of (PAA-*g*-PEG: 74.2%), (PAU-*g*-PEG: 70.4%) and (PAA: 51.6%). The two electrodes fabricated from PEG-grafted copolymers, PAA-*g*-PEG and PAU-*g*-PEG, showed good Coulombic efficiency, higher than 70% (Fig. 4b). The PAA-*g*-PEG electrode had a slightly higher Coulombic efficiency than the PAU-*g*-PEG electrode due to differences in the activation of the active material, that is Si, arising from differences in electrolyte uptake.

To determine differences in the electrolyte uptake of the silicon electrodes in relation to binder type, the three electrodes were stored in an electrolyte for 48 h under the same conditions, and swelling ratios were measured based on the weight changes of each electrode. First of all, PAA showed the highest electrolyte uptake, due to the strong H-bonding of the carboxylic acid groups in PAA with the carbonate-based electrolyte [ethylene carbonate (EC) and ethyl methyl carbonate (EMC)]. The PAA-*g*-PEG electrode, on the other hand, showed the lowest electrolyte uptake, due to its weakest H-bonding, caused by the grafting of PEG onto PAA. The PAU-*g*-PEG electrode, whose reduced hydrogen bonding interactions by PEG-grafting were compensated by UPy molecules, showed a higher electrolyte uptake (Figure S6).

It was, therefore concluded that the Si@PAA-*g*-PEG electrode had a relatively low lithiation capacity because of the low electrolyte uptake of this binder (PAA-*g*-PEG), which limits the activation of the active material in its initial cycle. The Si@PAU-*g*-PEG electrode, with its high electrolyte update, had a high lithiation capacity (Fig. 4c).

Afterwards, the Si@PAU-*g*-PEG electrode reached a high Coulombic efficiency of 94.0% in just two cycles, higher than that of PAA-*g*-PEG (92.6%) and PAA (81.1%). After the initial capacity fading, it quickly stabilized, and a Coulombic efficiency higher than 99% was maintained throughout. After 350 cycles, the Coulombic efficiency remained at a high of 99.4% (Fig. 4b). Overall, the Si@PAU-*g*-PEG electrode showed outstanding initial activation of silicon, and achieved high reversible capacity by overcoming the physical stress caused by volumetric change, through the stabilized ion conductive pathway.

**Figure 5:**
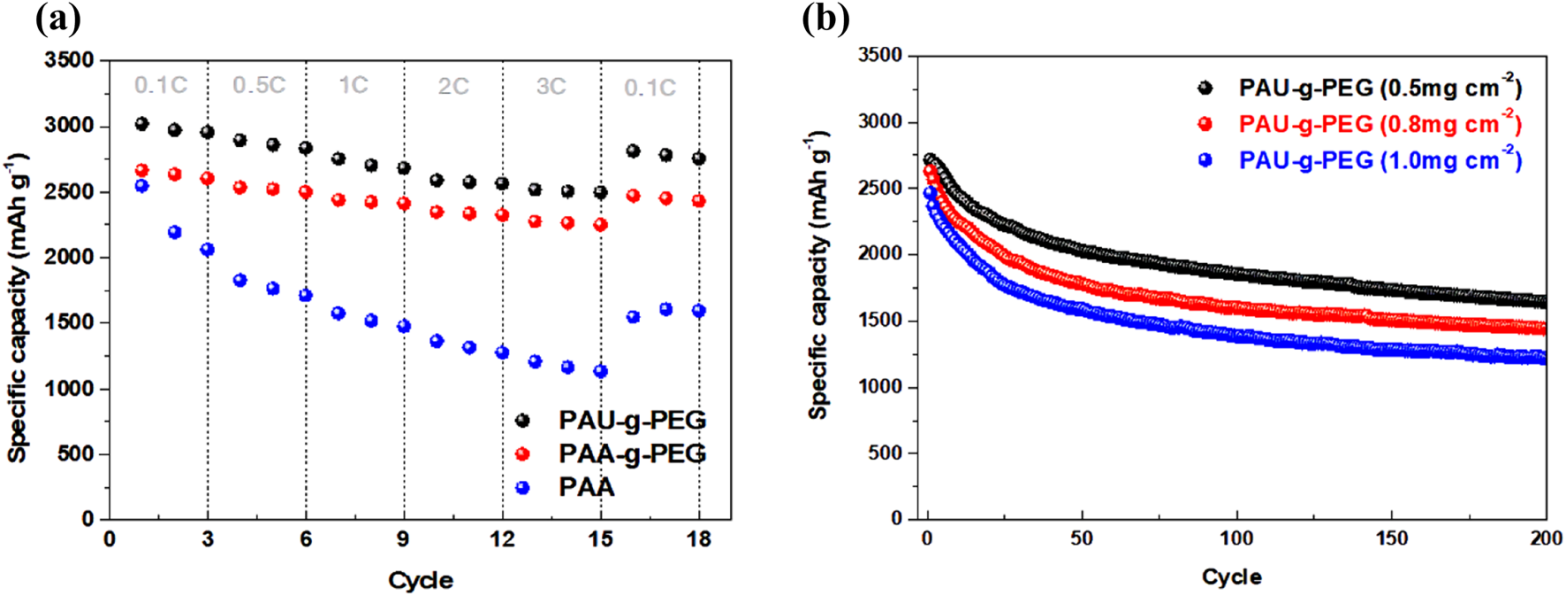
Electrochemical performance of the silicon electrodes using PAU-*g*-PEG, PAA-*g*-PEG and PAA as the polymeric binder: (**a**) rate performance at various current densities (@ 0.1–3 C), and (**b**) cycling performance (@ 0.5 C) of Si@PAU-*g*-PEG electrode having different mass loadings (0.5–1.0 mg cm^−2^).

Next, a C-rate performance test was conducted to determine rate capability under high current densities (Fig. 5a). The current density was fixed at 0.1 C for charging, and varied from 0.1 to 3 C for discharging.

The C-rate performance test revealed that the two electrodes fabricated from PEG polymers (PAU-*g*-PEG and PAA-*g*-PEG) had high rate capabilities. In particular, the Si@PAU-*g*-PEG electrode with the best cycling performance achieved a high capacity of 2,753, 2,590 and 2,519 mAh g^−1^ even under a high current density of 1, 2 and 3 C, respectively. When the current density was dropped to 0.1 C again, it demonstrated excellent rate capability, with a recorded capacity of 2,800 mAh g^−1^, which is close to its initial capacity of 2,900 mAh g^−1^.

The Si@PAA-*g*-PEG electrode also showed good rate capability and recovery (although not better than the PAU-*g*-PEG electrode) under all C-rate conditions. From these results, we can conclude that grafting PEG onto the PAA played a key role in enhancing ionic conductivity, and in turn, rate capability.

Compared with the recently reported polymer binders for silicon anodes, the electrode made of PAU-g-PEG showed moderate capacity at relatively high C-rate and cycle numbers, and excellent rate performance (Table S2).

The Si electrodes using the PAU-*g*-PEG binder were also prepared with various mass loading conditions of the active material, and their capacity retention was compared, to further evaluate the electrochemical properties of the Si@PAU-g-PEG, which exhibited the best cycle and rate characteristics (Fig. 5b). This was performed at high loading levels of 0.8 mg cm^−2^ and 1.0 mg cm^−2^, higher than the condition of 0.5 mg cm^−2^ at which stable cycle performance was obtained. The evaluation was conducted at 0.5 C for all three cells.

Although capacity decreased with increasing Si loading level, the initial capacity fading quickly stabilized for all three electrodes, and they subsequently showed good capacity retention. The electrodes fabricated with mass loadings of Si of 0.8 mg cm^−2^ and 1.0 mg cm^−2^ at 200 cycles maintained a capacity higher than 1,400 mAh g^−1^ and 1,200 mAh g^−1^ respectively. These results indicate that PAU-*g*-PEG binders can be utilized in silicon electrodes even at high loading levels, and that such electrodes are suitable for batteries with high energy densities.

The cycling performance of the Si@PAU-g-PEG electrode with low binder content (10 wt%) was also obtained at 0.5 C (Figure S7). The capacity and capacity retention of this electrode at 100 cycles were 1,333.3 mAh g^−1^ and 63.3%, respectively, indicating that the PAU-g-PEG binder showed good performance even with a high active material and a low binder content.

**Figure 6:**
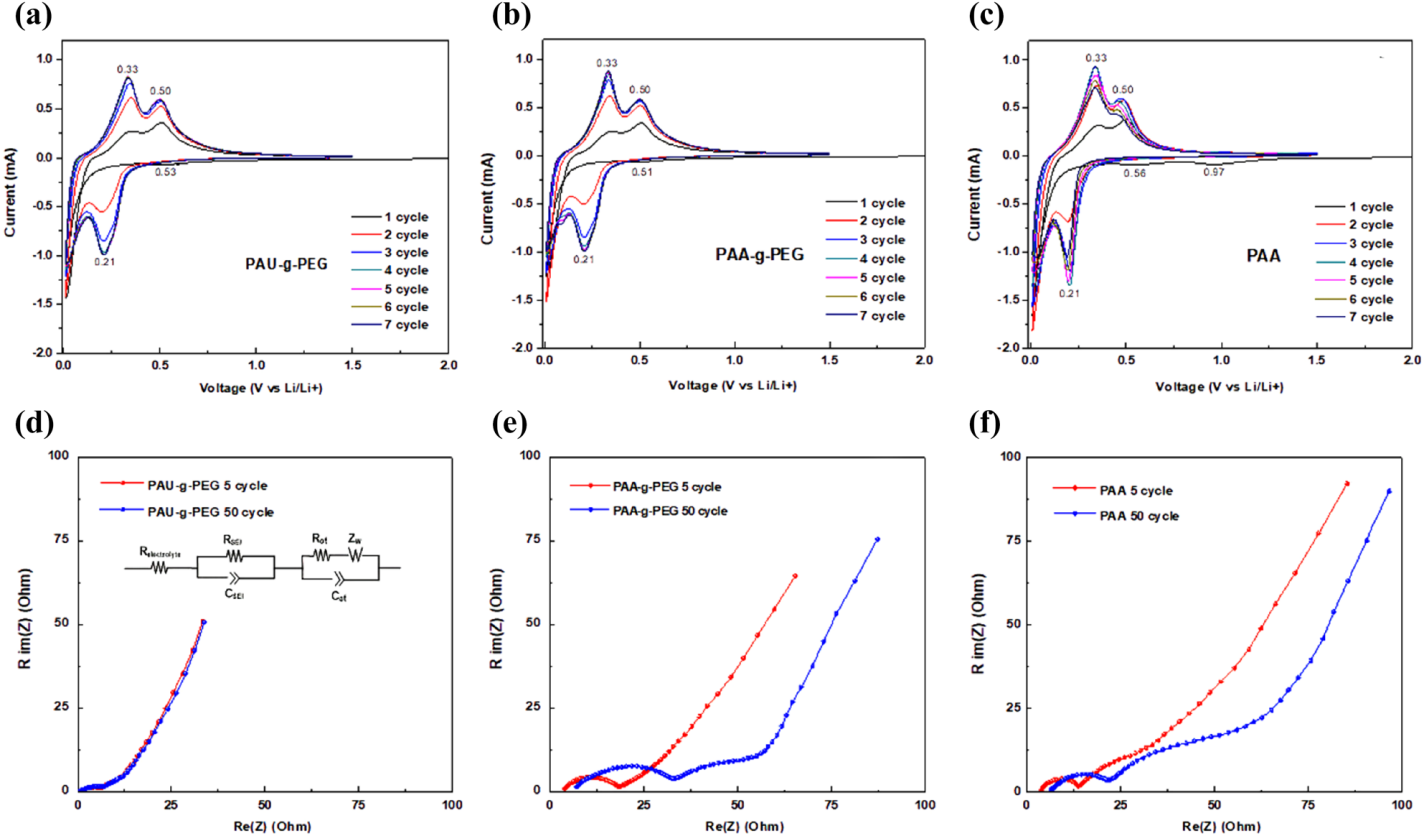
Electrochemical properties of the silicon electrodes using PAU-*g*-PEG, PAA-*g*-PEG and PAA as polymeric binders: (**a**–**c**) cyclic voltammetry curves and (**d**–**f**) electrochemical impedance spectroscopic graphs.

Next, cyclic voltammetry (CV) and electrochemical impedance spectroscopy (EIS) measurements were carried out to examine differences in the electrochemical properties of the silicon electrodes (Si@PAU-*g*-PEG, Si@PAA-*g*-PEG and Si@PAA in relation to their binder type (Fig. 6).

CV measurements were taken for all three electrodes under the same voltage range and scan rate conditions, and EIS measurements were obtained under the same charge/discharge conditions (0.5 C) and frequency range. The CV measurements showed that all three electrodes reached a cathodic peak at 0.21 V, corresponding to the lithiation of Si, and anodic peaks at 0.33 V and 0.50 V, corresponding to the delithiation process. All electrodes also experienced reversible electrochemical reactions (Fig. 6a–c). In addition, irreversible peaks were initially observed for all three electrodes in the range of 1.0–0.5 V due to the formation of an SEI layer in the cathodic scan domain of the first cycle, but such peaks were not seen in the second cycle. This means that side reactions did not occur in the charge/discharge voltage range, which verifies the electrochemical stability of the UPy-functionalized and PEG-grafted PAA, that is PAU-*g*-PEG, as a polymeric binder for silicon anodes.

Next, EIS measurements were taken for the three electrodes during the 5th and 50th charge/discharge process under a current density of 0.5 C after three formation cycles at 0.1 C. The first semi-circle represents the resistance (R_SEI_) due to the SEI layer, and the second semi-circle represents the resistance (R_CT_) due to charge transfer (Fig. 6d–f)^57,58^.

The results showed that both electrodes fabricated from the PAA-*g*-PEG and PAA binders showed an increase in R_SEI_ after cycles, but the size of the semi-circle was larger for the Si@PAA-*g*-PEG electrode, which also had a higher R_SEI_ after both 5 cycles and 50 cycles than the Si@PAA (Fig. 6e,f). This means that the silicon electrode with the PAA-*g*-PEG binder, which had the weakest physical stability, formed a thick SEI layer, as it was unable to tolerate the stress caused by volumetric expansion during the charge/discharge process.

As for R_CT_, the Si@PAA-*g*-PEG electrode revealed a lower resistance value than Si@PAA due to the enhanced lithium ion conductivity provided by the PEG domain. R_CT_ is influenced by both electronic and ionic resistance, since charge transfer involves the transfer of both electrons and lithium ions, and the electrode made of the binder (PAA-*g*-PEG), which had poor mechanical properties, lost electronic and ionic contact after cycling, leading to an increase in R_CT_^38,59^.

The Si@PAU-*g*-PEG electrode, which had both ionic conductivity and self-healing ability, was found to have much lower R_SEI_ and R_CT_ values, and maintained an impedance similar to the 5 cycles level, even after 50 cycles (Fig. 6d). This can be attributed to the formation of a strong electrical network between active materials due to the excellent in-electrode mechanical stability of the PAU-*g*-PEG binder, and low resistance due to the formation of ion transport channels by PEG. In particular, these results are attributed to the effective accommodation of physical stress produced by the volume expansion of Si, by introducing the self-healable Upy unit into the polymer binder.

Overall, the PAU-*g*-PEG binder alleviated the increase in resistance, by continuously recovering the structural collapse of the Si electrode, and also by maintaining electrical contact between active materials. These results are consistent with the good electrochemical performance data exhibited by the Si@PAU-*g*-PEG electrode.

### Lithium ion diffusion rate by polymeric binders

In addition, the lithium ion diffusion coefficients (D_Li+_) of the three electrodes (Si@PAU-*g*-PEG, Si@PAA-*g*-PEG and Si@PAA) were calculated and compared using the Warburg factor values obtained from the EIS measurements and GITT method of their formation cycles (Figure S8).

The calculated D_Li+_ from the EIS measurements was 8.02 × 10^–10^ cm^−2^ s^−1^, 3.88 × 10^–10^ cm^−2^ s^−1^, and 3.49 × 10^–11^ cm^−2^ s^−1^ for Si@PAU-*g*-PEG, Si@PAA-g-PEG and Si@PAA, respectively. That is, the Si@PAA-*g*-PEG electrode had a lithium ion diffusion coefficient ten times higher than that of the PAA binder electrode, which is an indicator of the lithium ion conductivity of PEG in the polymeric structure.

Additionally, the average value of lithium ion diffusion coefficients during charging was calculated from the GITT method^60^. The average D_Li+_ was 5.81 × 10^–12^ cm^−2^ s^−1^, 3.88 × 10^–12^ cm^−2^ s^−1^ and 3.05 × 10^–13^ cm^−2^ s^−1^ for Si@PAU-*g*-PEG, Si@PAA-*g*-PEG and Si@PAA. Although there are some differences in the values from GITT compared to those from EIS, the trend was the same.

Here, it should be noted that the Si@PAU-*g*-PEG electrode, comprised of both PEG and self-healable UPy unit, had a lithium ion diffusion coefficient two times higher than that of the Si@PAA-*g*-PEG electrode. This can be traced to the excellent mechanical stability of the Si@PAU-*g*-PEG electrode, as verified in the earlier peel-off test. That is, the physical crosslinking through the UPy moiety not only formed a more robust 3D network, but also helped to maintain this high structural stability during the charge/discharge cycles. As a result, the lithium ion pathway formed by the grafting of PEG was also well maintained, indicating that the Si@PAU-g-PEG electrode had higher ion conductivity than the Si@PAA-g-PEG electrode.

### Morphological analysis of the silicon electrodes

**Figure 7:**
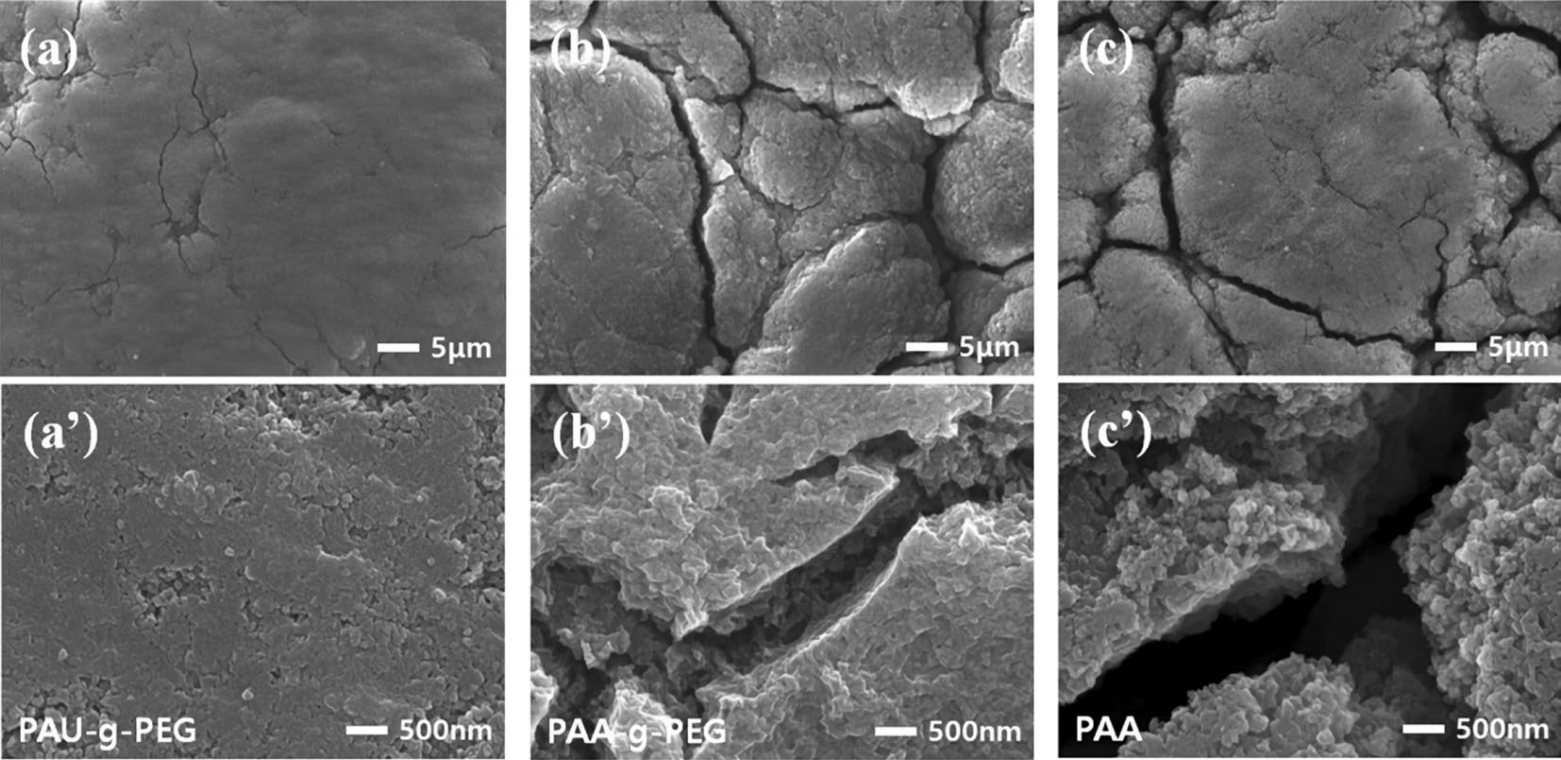
SEM images of the silicon electrodes after 50 cycles: (**a**) Si@PAU-*g*-PEG and (**a**′) its expanded form, (**b**) Si@PAA-*g*-PEG and (**b**′) its expanded form and (**c**) Si@PAA and (**c**′) its expanded form.

Morphological changes in the silicon electrodes after cycling were observed by scanning electron microscope (SEM) analyses (Fig. 7). The silicon electrodes fabricated from the three types of binders (PAU-*g*-PEG, PAA-*g*-PEG and PAA) all showed uniform morphologies before cycling, demonstrating the binders were well dispersed, due to interactions between the PAA main chain and silicon particles (Figure S10). After 50 cycles, however, very different morphologies were observed in the three electrodes.

At first, while slight cracks were seen in the Si@PAU-*g*-PEG electrode after 50 cycles, it was found that the morphology did not change much compared to that before cycling (Figure S10a and Fig. 7a).

Both the Si@PAA-*g*-PEG and Si@PAA electrodes, on the other hand, not only experienced severe cracking after 50 cycles, but also displayed non-uniform morphologies (Fig. 7b,c). This phenomenon is more apparent in their magnified images. The Si@PAU-*g*-PEG electrode maintained a uniform SEI layer, whereas the Si@PAA-*g*-PEG and Si@PAA electrodes formed thick, non-uniform SEI layers (Fig. 7a′–c′).

Furthermore, thickness changes after formation cycles were analyzed through cross-sectional SEM images (Figure S11). The thickness increase of Si@PAU-g-PEG, Si@PAA-g-PEG and Si@PAA electrodes was 8.57%, 19.0% and 31.4% respectively, when compared to thickness of the pristine electrodes. It can be seen that PAU-g-PEG binder showed the lowest thickness increase and maintained the good contact with the current collector, indicating excellent structural stability of electrode.

The above SEM results indicate the advantages of the PAU-*g*-PEG binder, which was capable of self-healing and had high physical stability due to the formation of a stable 3D network through physical crosslinking. These morphological properties were consistent with the results of the cyclability test and EIS, in which Si@PAU-*g*-PEG had the most outstanding capacity retention and lowest resistance.

## Discussion

In conclusion, this study employed simple radical copolymerization to develop a water-soluble copolymer PAU-*g*-PEG with self-healing ability and ionic conductivity, and used it as a polymeric binder for new silicon anodes. PEG was grafted onto the proposed PAU-*g*-PEG polymeric binder to achieve lithium ion conductivity, and this was verified through electrochemical impedance spectroscopy (EIS). A hydrogelation test demonstrated that the UPy molecules engaged in dynamic hydrogen bonds, inducing self-healing. The results of the Warburg impedance test revealed that the Si@PAU-*g*-PEG electrode, with UPy unit as a crosslinking site, formed a stable 3D network within the silicon electrode, and maintained stable lithium ion pathways even after cycling.

The silicon electrode with the PAU-*g*-PEG copolymer as binder retained a capacity of 1,450 mAh g^−1^ at 350 cycles and a Coulombic efficiency of 99.4% even after 350 cycles, as well as a high capacity of 2,500 mAh g^−1^ under a high current density of 3 C.

Based on the above, this study proved that the newly developed UPy-functionalized-PEG-grafted PAA, PAU-g-PEG, binder designed to have mechanical stability, self-healing ability and ionic conductivity was able to improve the electrochemical performance of silicon anodes, which previously experienced various issues due to severe volumetric change during charge/discharge cycles. These findings present new possibilities for multi-functional polymeric binders in lithium ion batteries with high energy densities.

## Methods

### Materials

Poly(acrylic acid) (PAA, *M*_*w*_: 100,000 g mol^−1^, 35 wt% in H_2_O), 2-amino-4-hydroxy-6-methylpyridine, *tert*-butyl acrylate (tBA) and poly(ethylene glycol) methyl ether methacrylate (PEG-MEMA) were obtained from Sigma-Aldrich. Hexamethylene diisocyanate, 2-hydroxyethyl acrylate (HEA) and dibutyltindilaurate (DBTDL) were purchased from TCI. Azobisisobutyronitrile (AIBN), hexane, chloroform, dimethylformamide, acetone, pyridine and trifluoroacetic acid (TFA) were received from Daejung. Silicon powder (crystalline, APS ≤ 50 nm) was purchased from Alfa-Aesar. The electrolyte (1 M LiPF_6_ in a combination of ethylene carbonate (EC) and ethyl methyl carbonate (EMC) (EC:EMC = 1:2 v/v) with 10% fluoroethylene carbonate (FEC) was purchased from Panax Etec. 2-(((6-(3-(6-methyl-4-oxo-1,4-dihydropyrimidin-2-yl)ureido)hexyl)carbamoyl)oxy)ethyl acrylate (UPy-containing monomer, UPyA) was prepared following the literature procedure^61^. For polymerization, each monomer was subjected to an inhibitor removal process, and HPLC grade solvent was used.

### Synthesis of poly(acrylic acid-*co*-UpyA-*g*-PEG) (PAU-*g*-PEG) 1

*tert*-Butyl acrylate (6.26 g, 48.8 mmol), Upy-containing monomer (UPy) (0.1 g, 244 μmol) and poly(ethylene glycol) methyl ether methacrylate (0.61 g, 1.22 mmol), together with AIBN (32 mg, 195 μmol), were dissolved in DMF (15 mL). The solution was sealed in a Schlenk flask and heated at 70 °C for 3 h under N_2_ atmosphere and the reaction was terminated by freezing the flask using liquid nitrogen. The solution was then precipitated in water and the filtrate was washed with distilled water several times. The solid was collected, and dissolved in trifluoroacetic acid (TFA) and left to stir for 3 h to give a white solid. The obtained solid was washed with hexane and acetone several times before it was dried under vacuum to give the product as a white powder (3.80 g, 89.2%); ^1^H NMR (400 MHz, DMSO-*d*_*6*_) *δ* ppm 11.98–12.38 (70H, br signal, H_y_), 7.34 (1H, br signal, H_x_), 7.04 (1H, br signal, H_x_), 5.74 (1H, br signal, H_x_), 3.46–3.50 (36H, br signal, H_z_), 3.13–3.43, 2.44–2.53, 2.00–2.40, 1.15–1.85 (11H, br signal, 3H_x_ + 3H_y_ + 5 Hz); GPC (DMF, RI)/Da *M*_*n*_ 1.07 × 10^5^, *M*_*w*_ 1.93 × 10^5^ and *M*_*w*_/*M*_*n*_ 1.80.

### Synthesis of poly(acrylic acid-*g*-PEG) (PAA-*g*-PEG) 2

This is typically same as the preparation of PAU-*g*-PEG without using Upy-containing monomer (UPy). The product was obtained as a white powder (3.60 g, 86.5%); ^1^H NMR (400 MHz, DMSO-*d*_*6*_) *δ* ppm 12.12–12.25 (70H, br signal, H_x_), 3.46–3.50 (36H, br signal, H_y_), 3.17–3.44, 2.43–2.50, 2.00–2.32, 1.16–1.83 (8H, br signal, 3H_x_ + 5H_y_); GPC (DMF, RI)/Da *M*_*n*_ 9.17 × 10^4^, *M*_*w*_ 1.42 × 10^5^ and *M*_*w*_/*M*_*n*_ 1.55.

### Fabrication of the Si nanoparticle (SiNP) electrode

The electrodes having a composition of the silicon nanoparticle (50 nm):Super P:binder = 6:2:2 and 7:2:1 weight ratio were prepared as follows: At first, the polymer binders were dissolved in distilled water by simply mechanical mixing. Silicon nanoparticles and Super P were mixed in dry condition and then each polymer binder solution was added into it to form a homogeneous slurry. The slurry with controlled viscosity was coated onto Cu foil using a doctor blade and dried in an oven at 80 °C for 30 min. As-formed electrodes were compressed to improve packing density by using roll-press machine. After cutting the electrodes with a size suitable to make the electrochemical evaluating cell, drying was conducted under vacuum at 120 °C for 3 h to remove the remaining water completely. Finally, the electrodes with the target specific mass loading values (0.5–1.0 Si mg cm^−2^) were prepared for further electrochemical studies.

### Characterization and measurements

^1^H NMR spectra were measured on Agilent 400-MR (400 MHz) instrument using DMSO-*d*_*6*_ as an internal deuterium lock and also as a solvent. FT-IR spectra were recorded on a PerkinElmer Spectrum Two ATR instrument over the range of 4000–400 cm^−1^.

The apparent molecular masses of the synthesized polymer were determined by Gel Permeation Chromatograph (GPC) using two PL Gel 30 cm × 5 μm mixed columns at 30 °C running in DMF and calibrated against poly(methyl methacrylate) (PMMA) standards (*M*_*n*_ = 2000-10^6^ g mol^−1^) standards using a Knauer refractive index detector.

The electrolyte uptake of the silicon anode was evaluated by the electrolyte absorption test as follows: Dry silicon was initially weighed (W_before_), immersed in the electrolytes composed of ethylene carbonate (EC) and ethyl methyl carbonate (EMC) (EC:EMC = 1:2 v/v) with 10% fluoroethylene carbonate (FEC) at room temperature for 48 h, and then was weighed (W_after_) again after excess electrolyte was wiped from their surfaces. The electrolyte uptake was calculated using Eq. (1)

1 $$ {\text{U}} = \frac{{W_{after} - W_{before} }}{{W_{before} }} \times 100 \;(\% ). $$

To measure the adhesion force of the polymeric binder for silicon electrode, a 180° peel-off test was performed using Universal Testing Machine (Shimadzu, EZ-L). The fabricated electrodes having same thickness were cut to a rectangular shape and were attached to the electrodes using the 3 M tape (12 mm width). By pulling the tape at a constant displacement rate of 30 mm min^−1^, the applied force was measured as an adhesion force.

Scanning electron microscopy (SEM) was performed on JEOL JSM-7800F instrument using each electrode after washing with dimethyl carbonate and drying under vacuum.

The thermal stabilities of the polymer binders were analyzed by Thermogravimetric Analysis (TGA). All polymer binders were fully dried under vacuum at 80 °C overnight before measurements, and TGA was performed on a Scinco TGA-N 1500 instrument over range of 30–800 °C in a nitrogen flow at a scanning rate of 20 °C min^−1^.

### Electrochemical performance analysis

To evaluate the electrochemical properties of the silicon electrodes, 2032-type coin cells and 3-electrode cells were assembled in an Ar-filled glovebox, using porous polyethylene (Celgard 2400) as a separator, lithium metal disc as a counter electrode, and 1 M LiPF_6_ in ethylene carbonate and ethyl methyl carbonate (1:2 v/v) with 10% fluoroethylene carbonate as electrolytes. In addition, a piece of lithium metal was used as a reference electrode in case of 3-electrode cell. In order to measure the cyclability, a galvanostatic discharge–charge cycling was conducted in the voltage range of 0.01–1.5 V vs Li/Li^+^ using CPS-Lab battery cycler (Basytec) at 25 °C in a temperature-controlled chamber. An electrochemical potentiostat system (VSP, Bio-Logic) was used to take electrochemical impedance spectroscopy (EIS), cyclic voltammetry (CV) and galvanostatic intermittent titration technique (GITT) measurements. EIS was acquired over a frequency range of 10 MHz to 100 kHz with AC amplitude of 10 mV, and CV was performed by scanning voltage at a rate of 0.1 mV s^−1^. The GITT tests were carried out at low current of 0.05 C applied for 15 min for each Coulometric titration step. After each charging step, the cell potential was stabilized to OCV for 30 min.

## Supplementary information

Supplementary Information.
