# Supplementary material for: A conductive self healing polymeric binder using hydrogen bonding for Si anodes in lithium ion batteries
Source: Sci Rep. 2020 Sep 11;10:14966. doi: 10.1038/s41598-020-71625-3 (PMC7486292; doi:10.1038/s41598-020-71625-3)
Supplement: Supplementary file 1 — Supplementary Information. [file 41598_2020_71625_MOESM1_ESM.docx]

***Supporting Information for***

**A conductive self healing polymeric binder using hydrogen bonding for Si anodes in lithium ion batteries**

Jaebin Nam,^1,2^ Eunsoo Kim,^1,2^ Rajeev K.K.,^1,2^ Yeonho Kim,^1,2^ and Tae-Hyun Kim^1,2*^

^1^Organic Material Synthesis Lab. Department of Chemistry, Incheon National University

^2^Research Institute of Basic Sciences, Incheon National University, 119 Academy-ro, Songdo-dong, Yeonsu-gu, Incheon 22012, Korea

^*^Corresponding Author, Tel: +82-32-835-8232; Fax: +82-32-835-0762; E-mail: [tkim@inu.ac.kr](mailto:tkim@inu.ac.kr) (T.-H. Kim)


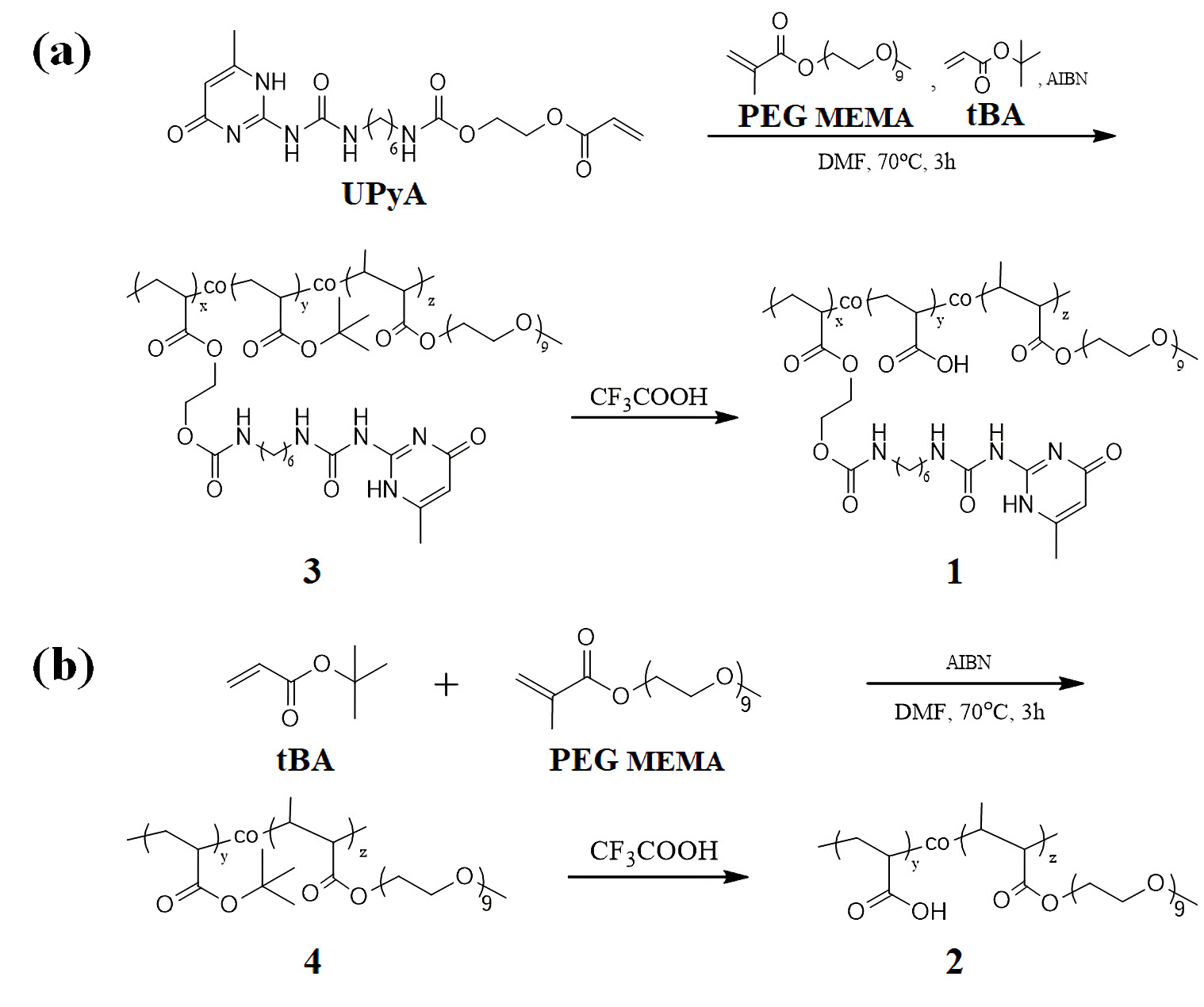


**Figure S1**. (a) Synthesis of PAU-*g*-PEG **1** and (b) its control polymer PAA-*g*-PEG **2**.


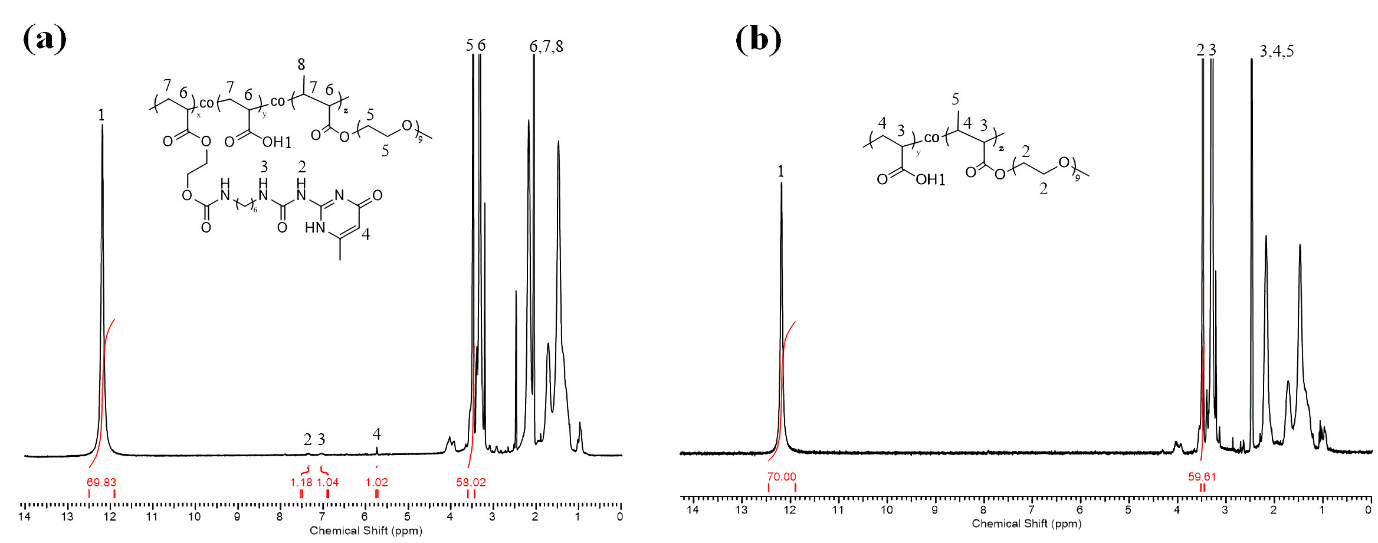


**Figure S2**. ^1^H-NMR spectra of the (a) PAU-*g*-PEG copolymer **1** and (b) PAA-*g*-PEG copolymer **2**.


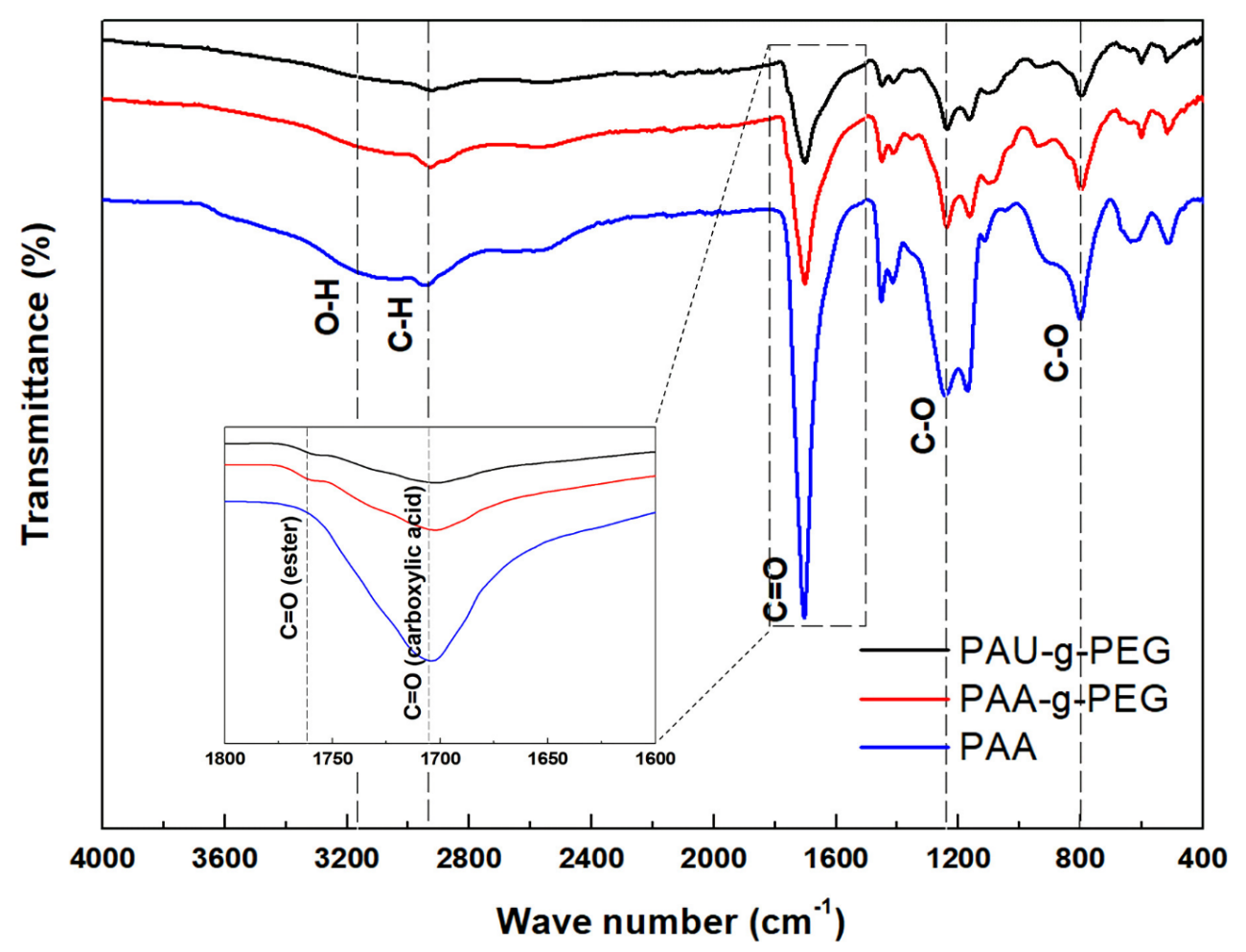


**Figure S3**. FT-IR spectra of PAU-g-PEG, PAA-g-PEG and PAA binder.


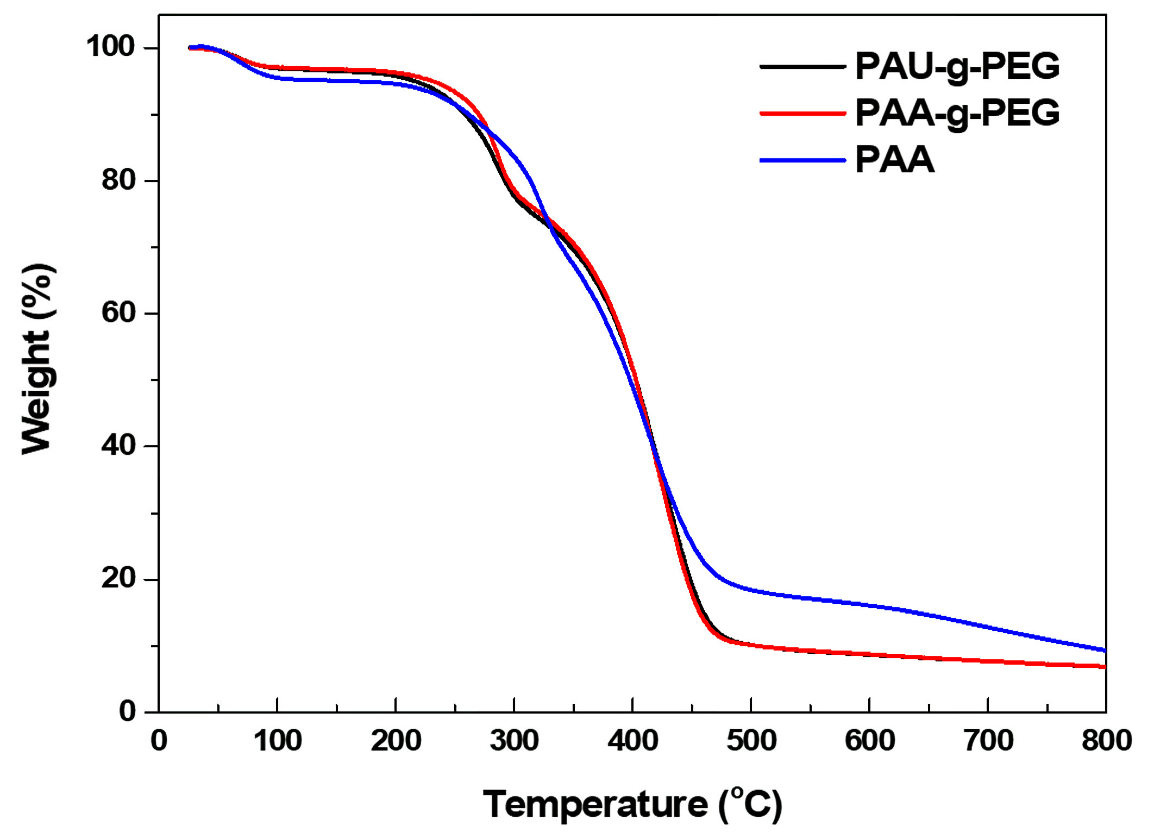


**Figure S4**. TGA thermograms of PAU-g-PEG, PAA-g-PEG and PAA binder.


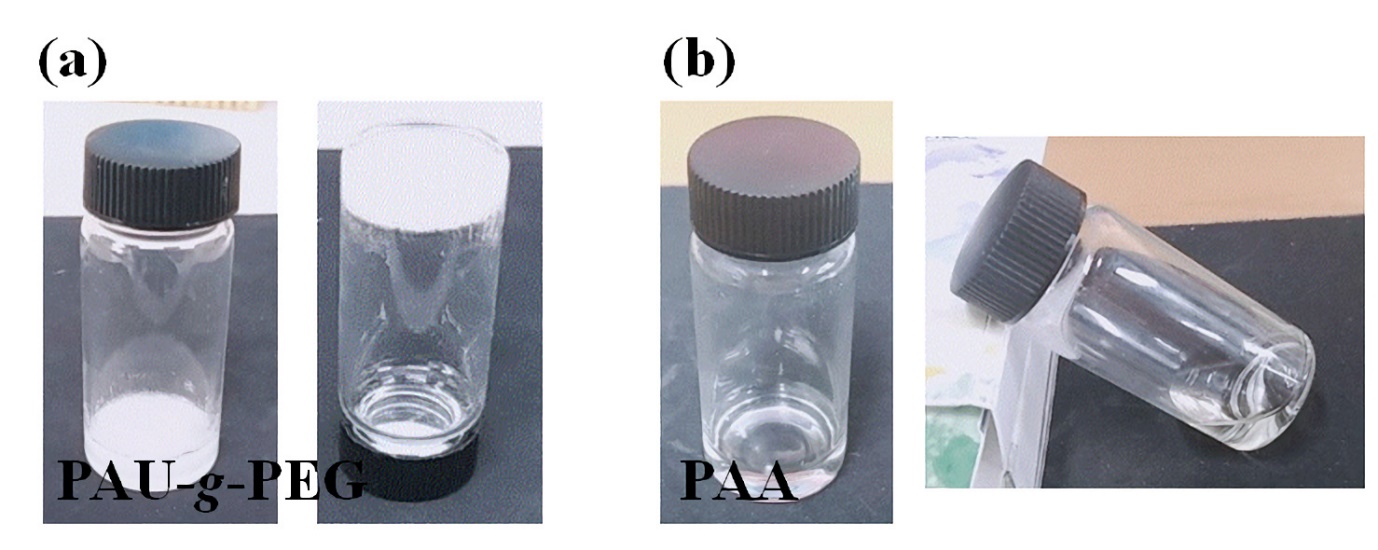


**Figure S5**. Photographs of the hydrogel (35 wt%) after the freeze/thaw process: (a) PAU-*g*-PEG and (b) PAA.


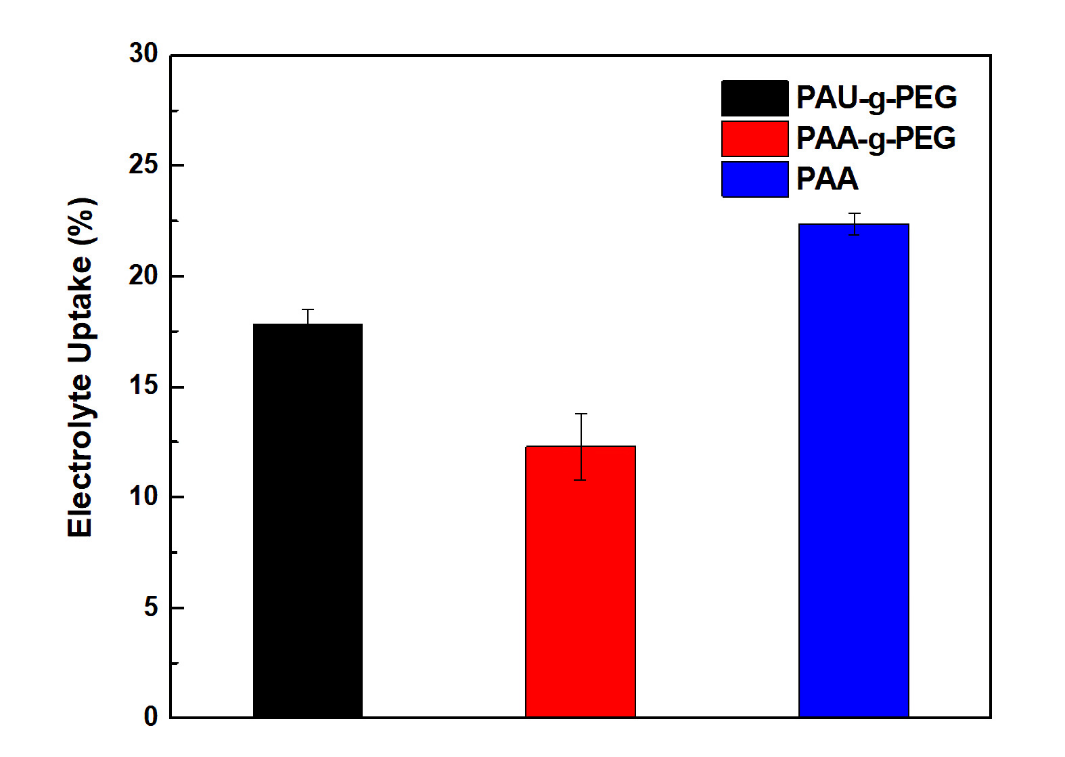


**Figure S6**. Electrolyte uptake results after soaking in electrolyte for 48 h.


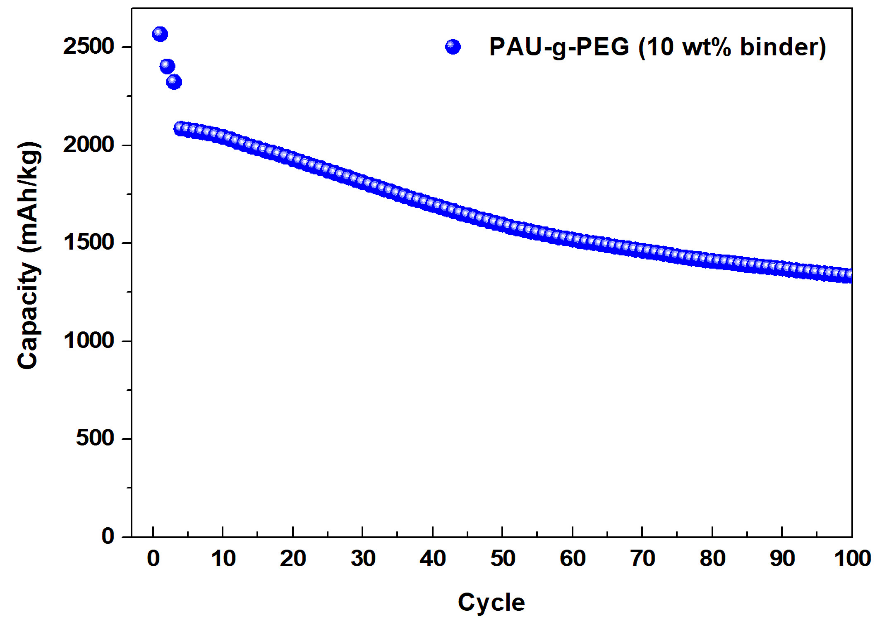


**Figure S7**. Cycling performance (@ 0.5 C) of the Si@PAU-g-PEG with low binder content (10 wt%).


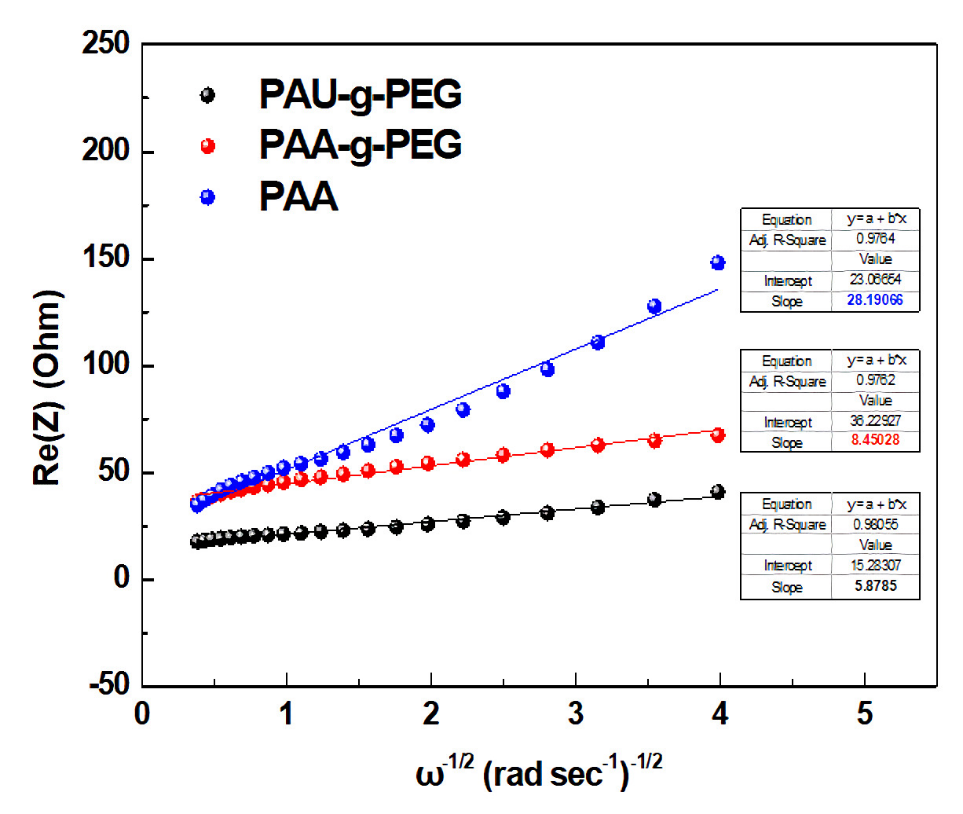


**Figure S8**. Warburg impedance plot at low frequency for the Si@PAU-*g*-PEG, Si@PAA-*g*-PEG and Si@PAA electrodes.


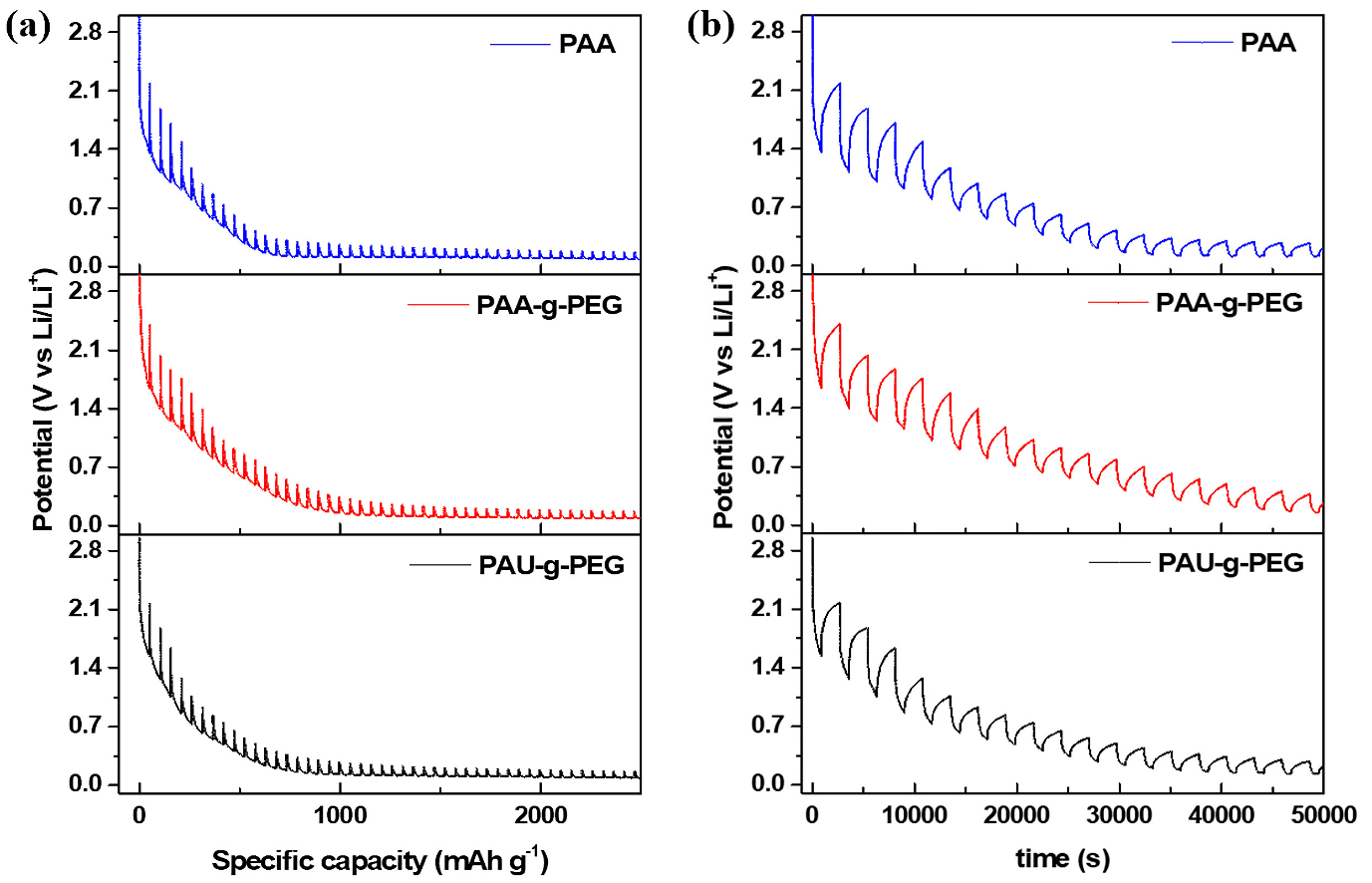


**Figure S9**. GITT curves for the first charging cycle for the Si electrodes: (a) voltage *vs*. capacity profiles and (b) voltage *vs*. time profiles.


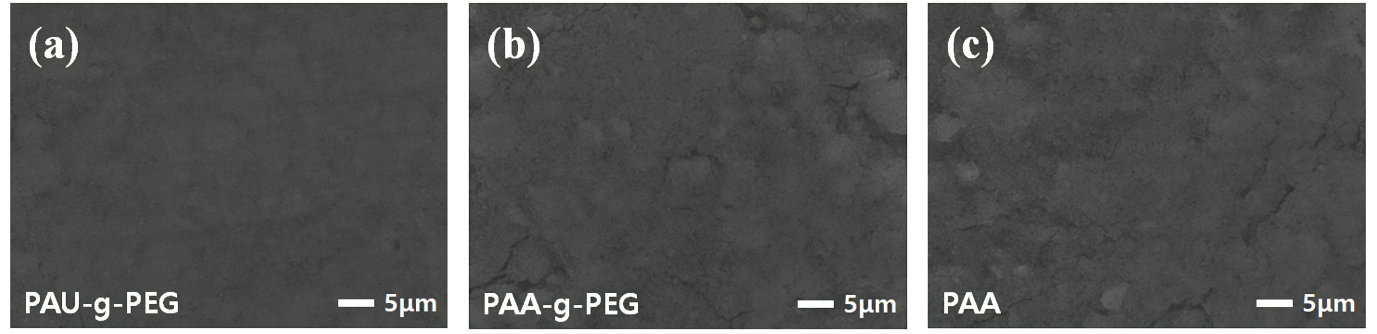


**Figure S10**. SEM images of the silicon electrodes before cycling: (a) Si@PAU-*g*-PEG, (b) Si@PAA-*g*-PEG and (c) Si@PAA.


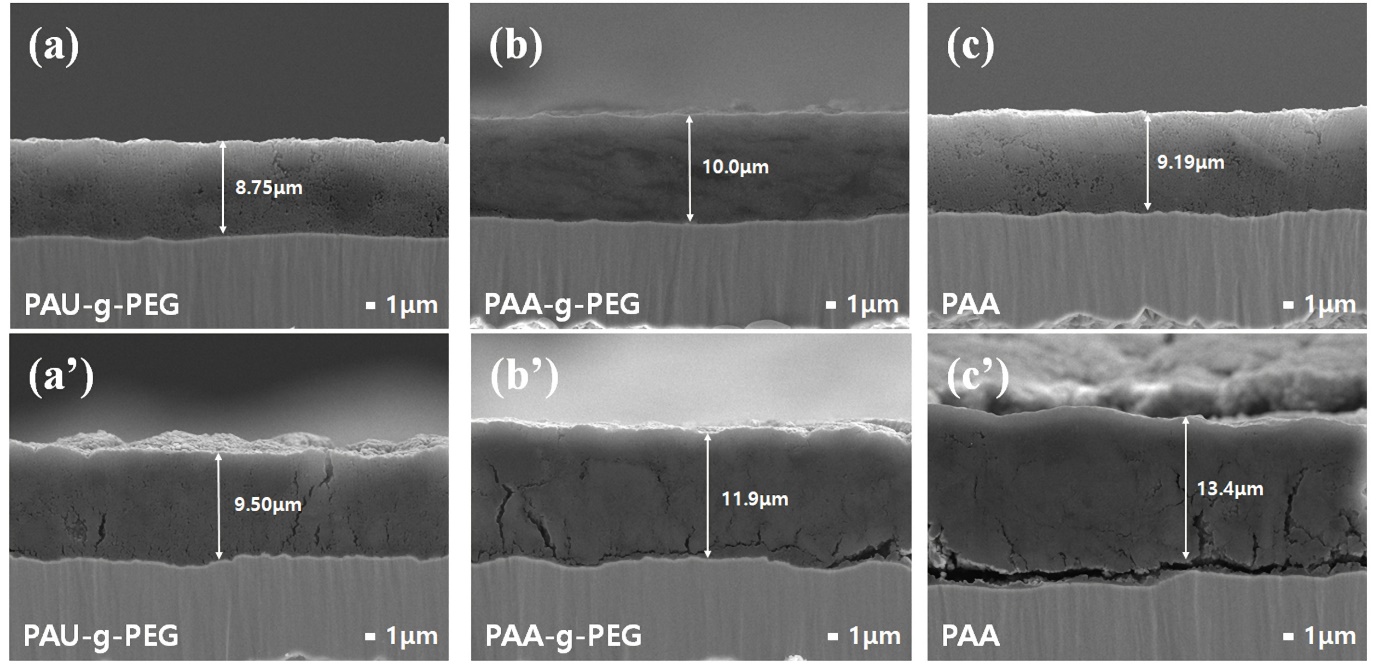


**Figure S11**. Cross-sectional SEM images of the silicon electrodes: (a) Si@PAU-g-PEG, (b) Si@PAA-g-PEG and (c) Si@PAA before cycling and (a’), (b’) and (c’) after formation cycles.

**Table S1**. GPC data and compositional analysis results of the copolymer **1** and its control polymer **2**.

| Polymer | M_n_ | M_w_ | PDI | Feed ratio | Actual ratio  (by ^1^H NMR) |
| --- | --- | --- | --- | --- | --- |
| PAU-*g*-PEG | 107 000 | 193 000 | 1.80 | 1 : 200 : 20 (Unit ratio of UPyA : tBA : PEG-MEMA) | 1 : 70 : 1.6 (Unit ratio of UPyA : AA : PEG-MEMA) |
| PAA-*g*-PEG | 91 700 | 142 000 | 1.55 | 200 : 20 (Unit ratio of tBA : PEG-MEMA) | 70 : 1.6 (Unit ratio of AA : PEG-MEMA) |

**Table S2**. Comparison of the electrochemical performances for Si-based electrodes.

| Type of binder | Electrode ratio  (Active material :  Conducting agent :  Binder) | Electrode mass loading  (mg cm^-2^) | Electrochemical performance | Ref |
| --- | --- | --- | --- | --- |
| PAU-*g*-PEG | 60: 20 :20 | 0.5 - 1.0 | 1450 mAh g^-1^ at 350th, 0.5 C  2500 mAh g^-1^ at 3 C (rate performance) | This work |
| PAA-5B | 60: 20 :20 | 1.2 – 1.5 | 1649 mAh g^-1^ at 100th, 0.5 C  1320 mAh g^-1^ at 1 C (rate performance) | 1 |
| s-PANI | 80 : 20  (without conducting agent) | 1.0 | 1776 mAh g^-1^ at 100th, 0.125 C  1583 mAh g^-1^ at 0.2 C (rate performance) | 2 |
| N-P-LiPN | 80 :10 : 10 | 0.7 – 1.0 | 1690 mAh g^-1^ at 100th, 0.5 C  2021 mAh g^-1^ at 2 C (rate performance) | 3 |
| PF-co-PDs | 80 : 2.5 : 17.5 | - | 1155 mAh g^-1^ at 500th, 0.1 C  225 mAh g^-1^ at 2 C (rate performance) | 4 |
| PF-c-PP/PVA | 60 : 20 : 20 | 0.7 - 0.75 | 2036 mAh g^-1^ at 100th, 0.33 C  - | 5 |
| PVA-PDA-PEDOT:PSS | 60 : 10 : 20 | 0.4 | 1600 mAh g^-1^ at 200th, 0.2 C  - | 6 |

References

1. Wang, S., Duan, Q., Lei, J., Yu, D. Y. W. Slime-inspired polyacrylic acid-borax crosslinked binder for high-capacity. *J. Power Sources* **468**, 228365, https://doi.org/10.1016/j.jpowsour.2020.228365 (2020).

2. Xiaoying, H. et al. Molecularly Engineered Conductive Polymer Binder Enables Stable Lithium Storage of Si. *Ind. Eng. Chem. Res.* **59**, 2680-2688, https://doi.org/10.1021/acs.iecr.9b05838 (2020).

3. Zeheng, L., Yaping, Z., Tiefeng, L. et al. Silicon Anode with High Initial Coulombic Efficiency by Modulated Trifunctional Binder for High-Areal-Capacity Lithium-Ion Batteries. *Advanced Energy Materials* **10**, 1903110, http://doi.org/10.1002/201903110 (2020).

4. Emrah, B., Emre. G., Neslihan, Y., Omer, S. T. Novel approach with polyfluorene/polydisulfide copolymer binder for high‐capacity silicon anode in lithium‐ion batteries. *J. Appl. Polym. Sci.* **136**, 48303 https://doi.org/10.1002/app.48303 (2020).

5. Omer, S. T., Neslihan, Y., Joan, P., George, A. J. Interconnected conductive gel binder for high capacity silicon anode for Li-ion batteries. *Materials Letters.* **273**, 127918, https://doi.org/10.1016/j.matlet.2020.127918 (2020).

6. Ruixian, T., Lei, M., Yu, Z. *et al.* A Flexible and Conductive Binder with Strong Adhesion for High Performance Silicon-Based Lithium-Ion Battery Anode. *ChemElectroChem* **7**, 1992-2000, https://doi.org/10.1002/celc.201902152 (2020).
